# Stranger Than Fiction

**Published:** 1893-03

**Authors:** 


### STRANGER THAN FICTION.

A photographer of Frankfort-on-the-Main, is responsible for the following narrative, published in the *Allgemeiner Anzeigeblat für Photographie* of that city : I have been a photographer for many years past. One day while I was eating my frugal meal, a very beautiful woman entered my studio, and wished to be photographed, because her husband strongly desired to possess her portrait. I immediately complied with her wish, and took her in various positions, but when I returned from the dark room, the lady had disappeared. The incident had an untoward appearance, and I feared I should lose by it. Nevertheless, I finished the portrait in the hope that the lady might, some day or another, return and pay me for it. And a few days afterward she did so. She admired the execution of it, although it appeared to me to be a little faint. At length she selected one of the copies, with these words : "Place this in your window, and write underneath it, 'Margaret Arlington.'" This surprised me, because, as you know, ladies do not like to have their portraits thus publicly exhibited ; and I concluded by supposing she was an actress. I thanked her, and she gave me a bank note for two pounds ten (fifty marks), and not having any change, I went into a chemist's on the ground floor, in order to obtain it, and give her the twenty-five shillings due to her. I placed the bank note in his hand, or at least I thought I did, but he asked, "Where is it?" It had disappeared. I searched upon the counter, and so did he and his assistant, but nothing could be found. I went back, examining the stairway, but no bank note was visible. What was I to say to the lady who was waiting above for her change ? I resolved to tell her what had happened, as perhaps, I might have received nothing. Entering the room I found the lady had disappeared, leaving five copies on the table. Here was a pretty state of things! At length I quieted myself with the thought that perhaps she was an actress, who was playing me this trick by way of advertising herself. At any rate I determined to exhibit the picture in the window. And I did well by it. Every day people came to sit, attracted by the photograph of the "beautiful blonde," as they called her, the story of which brought me in a good deal of money; so much so, that I would willingly have presented her with the five copies of it, and would have thanked her besides. Still I had a presentiment that I should, sooner or later, hear something of her.

A year after the occurrence, a gentleman in traveling costume entered my studio, and seemed pale and agitated. "There is," said he, "in your window, the photograph of a beautiful lady. Her name is Margaret Arlington, is it not?" "Yes," I replied, "that is her name." "Do you know the lady?" he asked. "Only from having photographed her. Perhaps you are acquainted with her?" I continued. "She is my wife; but I never knew anything of this photograph." "Thus it is," I rejoined, "the lady informed me that it would give her husband great pleasure to possess her portrait, as she had been for a long time separated from him." The gentleman turned pale, and trembling, asked, "When did this happen?" "A year ago," I answered. "My wife died five years ago." said the gentleman; "and you will perhaps doubt my sanity when I say that last night she appeared to me in a dream, saying, 'Go through the city; examine the windows of all the photographers, and you will find my portrait.' The dream was so real that I obeyed her, and thus have found her picture here."

I related to him all that had happened, and we were both convinced that the spirit of the lady had sat to me. I handed over to him the five copies, which appeared to me to be the best I had ever taken, and he insisted upon paying me for them. I refused, but he laid a bank note for £25 upon the table and quitted the room.

